# Fabrication of Carbamazepine Cocrystals: Characterization, *In Vitro* and Comparative *In Vivo* Evaluation

**DOI:** 10.1155/2021/6685806

**Published:** 2021-03-15

**Authors:** Muhammad Wasim, Abdul Mannan, Muhammad Hassham Hassan Bin Asad, Muhammad Imran Amirzada, Muhammad Shafique, Izhar Hussain

**Affiliations:** ^1^Department of Pharmacy, COMSATS University Islamabad, Abbottabad Campus Abbottabad 22060, Pakistan; ^2^Institute of Fundamental Medicine and Biology, Department of Genetics, Kazan Federal University, Kazan 420008, Russia; ^3^Department of Pharmaceutical Science, College of Pharmacy-Boys, Shaqra University, Al-Dawadmi Campus 17441, Shaqra 11911, Saudi Arabia

## Abstract

Carbamazepine (CBZ) is an antiepileptic drug having low bioavailability due to its hydrophobic nature. In the current study, efforts are made to investigate the effect of dicarboxylic acid coformer spacer groups (aliphatic chain length) on physicochemical properties, relative humidity (RH) stability, and oral bioavailability of CBZ cocrystals. Slurry crystallization technique was employed for the preparation of CBZ cocrystals with the following coformers: adipic (AA), glutaric (GA), succinic (SA), and malonic acid (MA). Powder X-ray diffractometry and Fourier-transform infrared spectroscopy confirmed cocrystal preparation. Physicochemical properties, RH stability, and oral bioavailability of cocrystals were investigated. Among the prepared cocrystals, CBZ-GA showed maximum solubility as well as improved dissolution profile (CBZ-GA > CBZ-MA > CBZ-AA > pure CBZ > CBZ-SA) in ethanol. Maximum RH stability was shown by CBZ-AA, CBZ-SA, and CBZ-MA. *In vivo* studies confirmed boosted oral bioavailability of cocrystals compared to pure CBZ. Furthermore, *in vivo* studies depicted the oral bioavailability order of cocrystals as CBZ-GA > CBZ-MA > Tegral® > CBZ-AA > CBZ-SA > pure CBZ. Thus, pharmaceutical scientists can effectively employ cocrystallization technique for tuning physicochemical properties of hydrophobic drugs to achieve the desired oral bioavailability. Overall, results reflect no consistent effect of spacer group on physicochemical properties, RH stability, and oral bioavailability of cocrystals.

## 1. Introduction

Pharmaceutical cocrystal (multicomponent system) consists of an active pharmaceutical ingredient (API) and coformer/cocrystal former [1]. The combination of chemically different components in a precise stoichiometric ratio leads to the formation of cocrystal [2]. The noncovalent interactions like *pi-pi* interactions, Van der Waals forces, and hydrogen bonding are predominantly present between the molecules of crystalline complex [3, 4]. Pharmaceutical cocrystallization is a good technique for enhancing the physicochemical properties of active pharmaceutical ingredients [5]. Physicochemical properties include solubility, dissolution, melting point, bulk density, hygroscopicity, and compressibility [1–3, 6]. Dissolution and solubility have primary significance owing to their principal role in drug oral absorption [7]. Many cocrystals have been synthesized for modification of physicochemical properties like posaconazole–4-aminobenzoic acid which exhibited high solubility and improved dissolution [8]. Similarly, improved dissolution rate and aqueous solubility were observed by carbamazepine–cinnamic acid cocrystal compared to pure carbamazepine (CBZ) [9]. Cocrystal temozolomide with baicalein enhanced oral drug bioavailability [10]. Carbamazepine–succinic acid cocrystal showed improved physicochemical properties and oral bioavailability [11]. Solubility, dissolution, and stability are the actual significant part of the cocrystal studies. Two factors determined the solubility: cocrystal components solvation and crystal lattice energy. Both factors can be influenced to various extents by cocrystallization [12, 13]. Over the last decade, different studies have been performed on many CBZ (BCS class II) cocrystals to increase its solubility. The low bioavailability of CBZ is due to poor solubility [14]. As stated by biopharmaceutical classification system (BCS), drugs having low solubility despite of high permeability are considered BCS class II drugs. These drugs have dissolution-restricted absorption and low oral bioavailability [15]. Therefore, to get the desired therapeutic effect, CBZ is used generally in a high dose [14].

**Figure 1:**
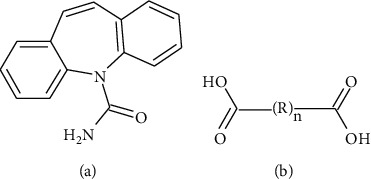
Chemical structures of (a) carbamazepine and (b) coformers (R-spacer group, *n* = 1, 2⋯).

Many cocrystals have been reported with a series of dicarboxylic acid coformers, for instance, itraconazole cocrystals with coformers like pimelic, adipic, glutaric, succinic, malonic, and oxalic acid; pyrazinecarboxamide with glutaric, succinic, and malonic acid coformers [13]; and CBZ cocrystals with malonic, succinic, glutaric, and adipic acid [16–18]. Different stoichiometry of CBZ cocrystal with coformers malonic (1 : 1; 2 : 1), succinic (2 : 1), adipic (2 : 1), and glutaric acid (1 : 1) has been reported [19]. Pyrazine was cocrystallized with a varying aliphatic chain length (spacer group) of dicarboxylic acid coformers to determine the influence on cocrystal formation and resulting structure of cocrystals [20]. If the spacer group of coformer changes, the conformation as well as the structure of crystal changes. Hence, self-assemblies formation by carboxylic acid coformers having flexible aliphatic spacer group cause changes in alignment and geometries of donors and acceptors [21]. Therefore, it is important to understand the influence of spacer group in dicarboxylic acid coformers (COOH)–(CH_2_)*_n_*–(COOH) (Figure 1): malonic (MA, *n* = 1), succinic (SA, n =2), glutaric (GA, *n* = 3), and adipic acid (AA, *n* = 4) on physicochemical properties and oral bioavailability of CBZ cocrystals.

## 2. Materials and Methods

### 2.1. Materials

CBZ was obtained from Tokyo Chemical Industry, Europe. The dicarboxylic acid coformers, MA, SA, and AA, were purchased from Acros Organics, while GA was procured from Sigma-Aldrich. All analytical grade solvents were purchased from commercial sources.

### 2.2. Cocrystal Preparation

Slurry crystallization method was used for bulk production of each cocrystal in different solvents. Solvents were added in screw-capped glass vials having solid, without reaching full dissolution. The mixtures were stirred for 72 hours at room temperature using a magnetic stirring bar. For further characterization, the resulting powder was rapidly filtered and dried. The detail is given in Table 1.

### 2.3. Characterization

#### 2.3.1. Powder X-Ray Diffractometry

Samples were characterized employing the XRPD method on a diffractometer (Siemens D5000). Samples were irradiated using Cu as the X-ray source at a current and voltage of 40 mA and 40 kV, respectively. A secondary monochromator was used to allow selection of the K*α* radiation of Cu (*λ* = 1.5418 Å). The samples were measured with a continuous scan rate of 0.01°/s from 2 to 50° at 2*θ*.

#### 2.3.2. Fourier-Transform Infrared Spectroscopy

An approximately 3–7 mg of samples was placed on crystal surface (diamond) to obtain the FT-IR spectra by using the PerkinElmer FT-IR spectrophotometer. The FT-IR spectral analysis of finely pulverized samples were carried out at a wavelength range of 450–4000 cm^−1^.

### 2.4. *In Vitro* Studies

*In vitro* studies like solubility and dissolution were performed for the prepared samples. Concerning dissolution experiments, the initial step was to grind CBZ cocrystals in mortar and pestle to obtain uniform particle size range. In the next step, the instrument (EasyMax 102 Advanced Synthesis Workstation) devised by Mettler Toledo provided with a temperature monitor and a stirrer rotating at 150 rpm to prepare supersaturated CBZ solution by pouring large quantity of sample powder in a flask (100 mL) having a medium (ethanol) of 40 mL at 25°C. Online “React IR iC 10” manufactured by Mettler Toledo AutoChem having an ATR crystal (AgX DiComp Fiber Conduit probe) with a size 6.5 mm attached to the crystallization reactor was used for recording of CBZ IR signature in solution every 15 seconds (50 scans) from 2800 cm^−1^ to 650 cm^–1^ till equilibrium. Finally, the React IR data was monitored using the “IR iC 10” software. The samples for solubility measurement were collected when the cocrystal solution reached the saturated state during dissolution experiment. Samples were analyzed by high-performance liquid chromatography (HPLC).

### 2.5. Relative Humidity Stability Study

Different relative humidity (RH) conditions like 43%, 75%, and 98% were obtained using salt solutions (saturated) of KCO_3_, NaCl, and K_2_SO_4_, respectively. The samples were kept in three different RH conditions for a period of 1 month. The samples were then immediately analyzed for absorption/adsorption of water by TGA-SDTA 851e devised by the Mettler Toledo thermogravimetric analysis (TGA) technique. The TGA thermograms were recorded at a temperature range of 30–150°C with a scanning rate of 10°C/min under a nitrogen purge of 50 mL min^–1^. The solid samples of masses around 8–10 mg were analyzed using aluminum oxide crucible. The STARe thermal analysis software was used for data evaluation.

### 2.6. Filling of Capsule Shells

Though capsule filling is a technical process, for research purposes, filling of capsules does not require specific machines due to small batch size. Therefore, all the suitable size capsules for *in vivo* pharmacokinetic studies were filled manually.

### 2.7. *In Vivo* Pharmacokinetic Studies

#### 2.7.1. Animals and Dosing

The protocols used for *in vivo* pharmacokinetic studies with the approval of “Research Ethical Committee” Department of Pharmacy, COMSATS University Islamabad (CUI), Abbottabad Campus (ref. no PHM-0088/E.C/M5). Healthy rabbits with a body weight 2 ± 0.3 kg were housed and restrained from food about 12 hours prior dosing while being allowed free access to water. All rabbits were randomly divided into five groups, each having six rabbits. Cocrystals and commercial product (pulverized) were filled into capsules and orally administered with 2 mL of water. Blood samples (0.5 mL) at different time intervals (0 to 18 h) were collected in heparinized tubes followed by separation of plasma by centrifugation for 10 min at 12000 rpm and then stored until further analysis at –20°C.

#### 2.7.2. Quantification of CBZ Plasma Concentration

CBZ was quantified in plasma samples as described previously [22] with slight modification, using the HPLC (series 200, PerkinElmer USA) technique. Mobile phase of acetonitrile, methanol, and phosphoric acid buffer (pH 6.5) 0.1 mol/L at a ratio of 10 : 30 : 60 was used. The flow rate and retention time were 1.2 mL/min and 3.2 min, respectively. A 250 × 4.6 mm Supelco® C_18_ (5 *μ*m particle size) was used as an analytical column. Acetonitrile was added to samples and centrifuged to precipitate proteins. A 20 *μ*L supernatant was injected. CBZ concentration was measured at *λ*_max_ 285 nm by UV detector.

#### 2.7.3. Data Analysis

Pharmacokinetic (PK) parameters like peak plasma concentration (*C*_max_) and time to reach peak plasma concentration (*T*_max_) were measured for noncompartmental model. Trapezoidal rule was employed for the calculation of area under curve (AUC_0→*t*_) from the concentration–time curve. Equation (1) was used for the calculation of total area under the curve (AUC_0→18_):

(1) $$\begin{array}{c} \text{AUC}0\to 18=\text{AUC}0\to 18+\frac{C\text{t}}{K\text{e}}, \end{array}$$

where *K*_e_ is CBZ elimination rate constant (apparent), *C*_t_ is CBZ concentration at 18th hour, and ANOVA (one-way analysis of variance) and *t*-test (*p* < 0.05) were used for comparison of PK parameters and statistical analysis of data.

## 3. Results

**Figure 2:**
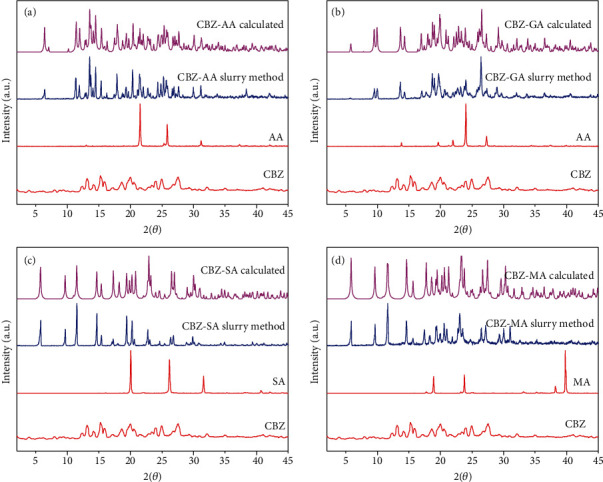
XRPD patterns of (a) CBZ-AA, (b) CBZ-GA, (c) CBZ-SA, and (d) CBZ-MA cocrystals.

**Figure 3:**
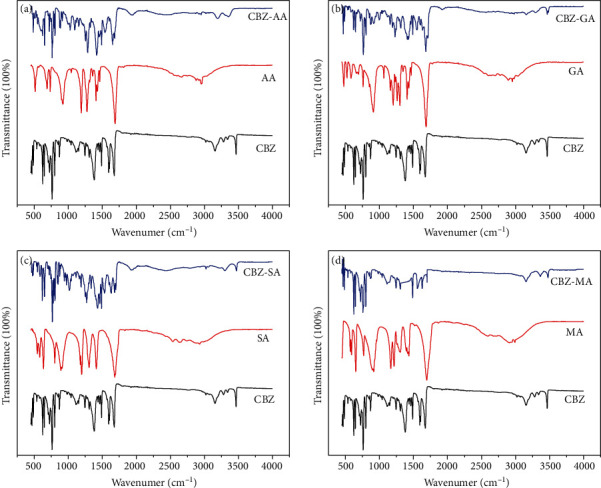
FT-IR spectra of (a) CBZ-AA, (b) CBZ-GA, (c) CBZ-SA, and (d) CBZ-MA cocrystals.

**Figure 4:**
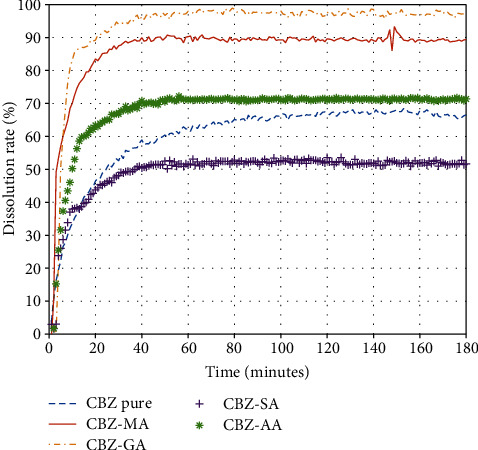
Dissolution release profile of CBZ-AA, CBZ-GA, CBZ-SA, CBZ-MA, and pure CBZ in ethanol.

**Figure 5:**
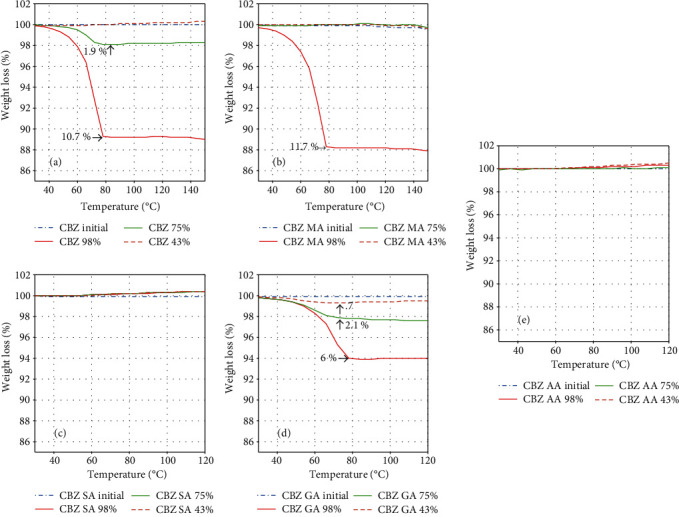
TGA thermograms of (i) pure CBZ, (ii) CBZ-MA, (iii) CBZ-SA, (iv) CBZ-GA, and (v) CBZ-AA.

**Figure 6:**
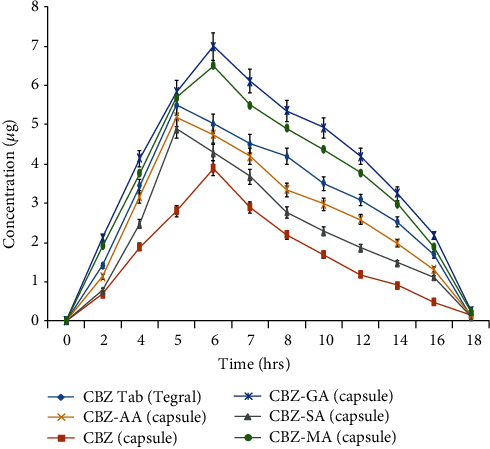
*In vivo* drug release profile of pure CBZ, cocrystals, and marketed product.

The powder XRPD patterns of cocrystals are different from its individual components, i.e., CBZ and respective coformers. The XRPD patterns of CBZ-AA, CBZ-GA, CBZ-SA, and CBZ-MA are perfectly matched with their XRPD powder patterns provided in the Cambridge Structural Database (CSD) as MOXVEB, MOXVOL, XOBCIB, and XOBCEX, respectively (Figure 2). FT-IR spectra of cocrystals are different from its individual components, i.e., pure CBZ and respective coformers as shown in Figure 3. *In vitro* studies, like solubility and dissolution behavior of pure CBZ and its four cocrystals, were investigated. The dissolution of cocrystals was improved except CBZ-SA compared to pure CBZ as shown in Figure 4. Cocrystals showed better dissolution whose solubility was enhanced by cocrystallization. The result is summarized in Table 2. The results of RH stability studies of pure CBZ show a weight loss of 10.7% and 1.9% at 98% and 75% RH conditions, respectively. CBZ-AA and CBZ-SA showed maximum stability with no weight loss. CBZ-GA, the most unstable, even showed a weight loss at 43% RH as shown in Figure 5 and Table 3. A probe into the *in vivo* pharmacokinetic study was conducted in rabbits. Plasma drug profile of pure CBZ, cocrystals, and marketed product is shown in Figure 6. Significant increase in oral bioavailability was observed with cocrystals compare to pure CBZ. CBZ-GA exhibited the highest peak plasma concentration (7 ± 0.35 *μ*g/mL) with high oral bioavailability (64 ± 2.2 *μ*g h/mL).  Table 4 enlists pharmacokinetic parameters.

## 4. Discussion

The characteristic XRPD peaks of polymorph CBZ-III^#^ (Figure S1), used in the study that appeared at 2*θ* = 15.8 and 18.6, were found to be in good agreement with reported data [23]. CBZ was cocrystallized with dicarboxylic acid coformers, i.e., AA, GA, SA, and MA to investigate the spacer group effects on physicochemical properties and oral bioavailability of cocrystals. The formation of cocrystals was confirmed by XRPD and FT-IR. The XRPD pattern of samples was in good agreement with their corresponding XRPD patterns in CSD. Figure 2 shows overlays of XRPD pattern of cocrystals consistent with the published data [24]. There are two reported polymorphs of CBZ-MA cocrystals with CSD codes XOBCEX (2 : 1) and MOXVUR (1 : 1) as shown in Figure S1 [19]; a third form has also been studied, but a single crystal was not yet obtained via solvent crystallization method [25]. CBZ-MA and CBZ-SA have similar packing of CBZ and hydrogen-bonding motif. So these two are isostructural [19]. Further validation of cocrystals was carried out by FT-IR spectral analysis. The FT-IR spectra of cocrystals are different from their respective components (Figure 3 and Table 5). The pure CBZ shows the absorption bands of N–H stretching and C=O stretching at 3465 and 1675 cm^−1^, respectively [26]. Clear shifts were observed in CBZ-AA, CBZ-GA, CBZ-SA, and CBZ-MA cocrystals due to hydrogen-bond formation. Thus, the XRPD and FT-IR analyses authenticate the formation of CBZ cocrystals with AA, GA, SA, and MA coformers.

*In vitro* results of CBZ cocrystals showed that about 60% of pure CBZ was dissolved in 45 min. But CBZ-SA cocrystal dissolved 52% in 45 min being the lowest soluble, whereas dissolution of CBZ-GA, CBZ-MA, and CBZ-AA was 97%, 90%, and 70%, respectively (Figure 4). CBZ-GA (1 : 1) showed highest solubility (24.92 ± 0.03 mg/mL), while CBZ-SA (2 : 1) cocrystal exhibited low solubility of 8.88 ± 0.01 mg/mL as shown in Table 2. Thus, the CBZ to coformer stoichiometry seems to have no effect on solubility as well as dissolution. The higher final concentration of CBZ was achieved by all cocrystals except CBZ-SA compared to pure CBZ. This might be due to CBZ-SA cocrystal being low soluble in ethanol [27]. The interesting thing is that maximum solubility of CBZ was observed in 40 minutes by all cocrystals, whereas approximately 100-minute longer time was needed for pure CBZ. In cocrystal, the more soluble component (coformer) is usually drawn out of the crystal lattice into dissolution medium [28]. The solubility of dicarboxylic acids series in different solvents including ethanol was determined by Zhang and coworkers to check the effect of “even–odd” number of carbons. The solubility of coformer having odd number of carbons was higher compared to coformer of even number of carbons [29]. Thus, the “even–odd” number of carbons of coformers may also affect the solubility of cocrystal. In our study results, it has been noted that cocrystals having coformer (odd number of carbons) showed higher solubility and vice versa (Table 2). Likewise, in the previous study, the solubility order of coformers (GA > MA > AA > CBZ-SA) in ethanol has been reported [29]. Our results of cocrystal solubility are consistent with the order of solubility of coformers. From a pharmaceutical point of view, a significant dissolution profile was observed by CBZ-GA being the most soluble released about 97% CBZ in dissolution medium. The enhanced solubility and dissolution of CBZ-GA in the presence of excess coformer is also reported [30]. Past studies also explored that dissolution and solubility can be improved by cocrystallization technique [8, 31–33]. Likewise, an improved dissolution rate by carbamazepine–cinnamic acid cocrystal was observed in distilled water compared to pure CBZ [9]. Therefore, enhanced dissolution of cocrystals can be attributed to solubility improvement in the dissolution medium.

Concerning RH stability studies, CBZ-GA showed % weight loss of 0.7, 2.1, and 6 at 43, 75, and 98% RH conditions, respectively, whereas CBZ-SA and CBZ-AA showed zero % weight loss at provided RH conditions. The weight loss of 11.7% was observed in CBZ-MA at 98% RH and remained stable at 43 and 75% RH. However, the trend of % weight loss in pure CBZ was 1.9 and 10.7 at 75% and 98% RH, respectively, while stable at 43% RH condition as shown in Figure 5 and Table 3. So, overall, CBZ-RH stability has been improved by cocrystallization. It was observed that CBZ-GA is the most unstable cocrystal with highest solubility and improved dissolution. It was also seen in the previous studies that a highly soluble cocrystal is least stable [34]. A pure CBZ and cocrystals were evaluated for oral bioavailability in rabbits along with marketed product to assess the spacer group effect and to confirm the *in vitro* improvement of CBZ cocrystals translation into *in vivo* pharmacokinetic benefit. The plasma drug concentration vs. time profile and pharmacokinetic parameters are given in Figure 6 and Table 4, respectively. The results of *in vivo* pharmacokinetic study showed that highest oral bioavailability (64 ± 2.2 *μ*g h/mL) and *C*_max_ (7 ± 0.35 *μ*g/mL) were observed by CBZ-GA. The oral bioavailability of CBZ-GA was significantly higher than pure CBZ (29.87 ± 2.1 *μ*g h/mL) and marketed product (56.03 ± 1.2 *μ*g h/mL). The AUC_0–*t*_ of CBZ-MA (58 ± 1.8 *μ*g h/mL) was not considerably higher than marketed product. Moreover, the bioavailability and *C*_max_ of prepared cocrystals were much higher than pure CBZ. But, no correlation was observed between oral bioavailability and spacer group of coformers. However, the *in vivo* study results exhibit that solubility and dissolution-restricted oral bioavailability can be enhanced by the cocrystallization technique.

## 5. Conclusion

The present research work concludes the influence of spacer group (varying aliphatic chain length) on solubility, dissolution, RH stability, and oral bioavailability of CBZ cocrystals with dicarboxylic acids coformers. According to the reported results, a good improvement in dissolution is shown by CBZ-GA being the most soluble and unstable cocrystal. The low soluble cocrystal is CBZ-SA which does not show improved dissolution. Hence, the order of enhanced solubility and dissolution is CBZ-GA (*n* = 3) > CBZ-MA (*n* = 1) > CBZ-AA (*n* = 4) > pure CBZ > CBZ-SA (*n* = 2). The highly and poorly soluble cocrystals show the highest and lowest dissolution, respectively. So, solubility and dissolution are consistent with each other. Similarly, the increased order of oral bioavailability observed is CBZ-GA (*n* = 3) > CBZ-MA (*n* = 4) > marketed product > CBZ-AA (*n* = 2) > CBZ-SA (*n* = 1) > pure CBZ. In conclusion, no consistency of spacer group effects is seen on physicochemical properties (solubility and dissolution) and oral bioavailability of CBZ cocrystals. In other words, increasing hydrophobicity of coformer has no regular effect on cocrystals physicochemical properties and oral bioavailability.

## Figures and Tables

**Table 1:** Complete detail about cocrystals regarding their preparation.

API	Coformer	Ratio	Solvent	Stirring time (h)
CBZ	AA	2 : 1	Methanol	72
CBZ	GA	1 : 1	Acetone	72
CBZ	SA	2 : 1	Ethanol	72
CBZ	MA	2 : 1	Methanol	72

Abbreviations: CBZ: carbamazepine, MA: malonic acid, SA: succinic acid, GA: glutaric acid, AA: adipic acid.

**Table 2:** Solubility and % dissolution rate about different cocrystals in ethanol.

S. No.	Samples	Cocrystal stoichiometry	Solubility (mg/mL)	% dissolution rate (45 min)
01	CBZ	—	18.19 ± 0.06	60
02	CBZ-MA	2 : 1	24.61 ± 0.08	90
03	CBZ-SA	2 : 1	8.88 ± 0.01	52
04	CBZ-GA	1 : 1	24.92 ± 0.03	97
05	CBZ-AA	2 : 1	22.62 ± 0.10	70

**Table 3:** The % weight loss of pure CBZ, CBZ-MA, CBZ-SA, CBZ-GA, and CBZ-AA.

Samples	% relative humidity	% weight loss
CBZ	43	—
	75	1.9
	98	10.7
CBZ-MA	43	—
	75	—
	98	11.7
CBZ-SA	43	—
	75	—
	98	—
CBZ-GA	43	0.7
	75	2.1
	98	6
CBZ-AA	43	—
	75	—
	98	—

**Table 4:** Pharmacokinetic parameters of pure CBZ, cocrystals, and marketed product.

Formulation	AUC_0–18_ (*μ*g h/mL)	*C*_max_ (*μ*g/mL)	*T*_max_ (h)
CBZ tab (marketed)	56.03 ± 1.2	5.5 ± 0.32	5 ± 0.72
CBZ (capsule)	29.87 ± 2.1	3.9 ± 0.27	6 ± 0.22
CBZ-SA (capsule)	47.81 ± 2.00	4.9 ± 0.31	5 ± 0.23
CBZ-AA (capsule)	50.17 ± 1.00	5.2 ± 0.21	5 ± 0.21
CBZ-GA (capsule)	64 ± 2.2	7 ± 0.35	6 ± 0.27
CBZ-MA (capsule)	58 ± 1.8	6.5 ± 0.30	6 ± 0.38

**Table 5:** FT-IR peaks summary of individual components and respective cocrystals.

Compounds	Peaks (cm^−1^)	Groups	Inference
CBZ	3465	N–H stretching	—
	1675	C=O stretching	
AA	1688	C=O stretching	—
GA	1686	C=O stretching	—
SA	1682	C=O stretching	—
MA	1696	C=O stretching	—
CBZ-AA	3355	N–H stretching	Cocrystal formed
	1698	C=O stretching	
CBZ-GA	3482	N–H stretching	Cocrystal formed
	1712	C=O stretching	
CBZ-SA	3469	N–H stretching	Cocrystal formed
	1698	C=O stretching	
CBZ-MA	3481	N–H stretching	Cocrystal formed
	1701	C=O stretching	

Abbreviations: CBZ: carbamazepine, MA: malonic acid, SA: succinic acid, GA: glutaric acid, AA: adipic acid.

## Data Availability

Data used to support this study finding have been included in the article and could be provided upon request from first author Muhammad Wasim (wassypharmacist@gmail.com).

## Supplementary Materials

Supplementary Materials Figure S1: it contains XRPD patterns of different forms of CBZ and CBZ-MA.
