# Supplementary material for: Fabrication of Carbamazepine Cocrystals: Characterization, In Vitro and Comparative In Vivo Evaluation
Source: Biomed Res Int. 2021 Mar 15;2021:6685806. doi: 10.1155/2021/6685806 (PMC7987437; doi:10.1155/2021/6685806)
Supplement: Supplementary Materials — Figure S1: it contains XRPD patterns of different forms of CBZ and CBZ-MA. [file 6685806.f1.docx]

**Supplementary Information**

**Fabrication of Carbamazepine Cocrystals: Characterization, *In-Vitro* and Comparative *In-Vivo* Evaluation**

**Muhammad Wasim^1^, Abdul Mannan^1^, Muhammad Hassham Hassan Bin Asad^1,2^, Muhammad Imran Amirzada, ^1^ Muhammad Shafique^3^, and Izhar Hussain^1^**

*^1^Department of Pharmacy, COMSATS University Islamabad, Abbottabad Campus 22060, Pakistan*

*^2^Institute of Fundamental Medicine and Biology, Department of Genetics, Kazan Federal University, Kazan 420008, Russia*

*^3^Department of Pharmaceutical science, College of Pharmacy-Boys, Al-Dawadmi Campus 17441, Shaqra University, Shaqra 11911, KSA*





F_IGURE_ S1: Comparison of XRPD patterns of CBZ-MA (MOXVUR, 1:1), CBZ-MA (XOBCEX, 2:1) and CBZ-MA obtained via slurry method.





F_IGURE_ S2: Comparison of XRPD patterns of different forms of CBZ. (^#^used in this research work)
